# End-tidal capnographic monitoring to detect apnea episodes during flexible bronchoscopy under sedation

**DOI:** 10.1186/s12890-016-0361-7

**Published:** 2017-01-07

**Authors:** Tsukasa Ishiwata, Kenji Tsushima, Mai Fujie, Kenichi Suzuki, Kosuke Hirota, Mitsuhiro Abe, Naoko Kawata, Jiro Terada, Koichiro Tatsumi

**Affiliations:** 1 Department of Respirology, Graduate School of Medicine, Chiba University, 1-8-1, Inohana, Chuo-ku, Chiba 260-8670 Japan; 2 Medical Equipment Control Center, Chiba University Hospital, 1-8-1, Inohana, Chuo-ku, Chiba 260-8670 Japan

## Abstract

**Background:**

Apnea developing as a result of oversedation is a potential clinical problem in patients undergoing flexible bronchoscopy (FB) under sedation. However, there are no reports of evaluation using a standardized method of the frequency of occurrence of apnea episodes during FB under sedation. The aim of this study was to investigate the frequency of apnea episodes during FB under sedation in the clinical setting by end-tidal capnography.

**Methods:**

This study was a single-institution retrospective review of a prospectively maintained database and medical records, including capnographic data, from April 2015 to March 2016. We enrolled patients who were sedated with midazolam and underwent diagnostic FB under end-tidal capnographic monitoring. Apnea was defined as cessation of airflow for more than 10 s.

**Results:**

Data from a total of 121 eligible patients were analyzed. A total of 131 apnea episodes (median duration 33 s) were recorded in 59 patients (48.8%). Prolonged apnea episodes lasting for more than 30 s occurred in 24 patients (19.8%). Furthermore, 55 apnea episodes (42.0%) were followed by a decline of the SpO_2_ by ≥4% from the baseline.

**Conclusions:**

In this study, end-tidal capnography revealed the occurrence of apnea episodes at a high frequency in patients undergoing FB under sedation in the clinical setting.

## Background

Sedation is required for relieving the anxiety and calming of patients during a wide variety of medical procedures [1]. However, inadvertent oversedation may sometimes occur, leading to deep sedation and apnea episodes [2]. Bronchoscopic procedures require moderate sedation to alleviate the patient’s anxiety and to suppress the cough reflex. However, this is associated with the risk of respiratory depression developing during prolonged procedures, especially diagnostic bronchoscopies, as administration of high doses of sedatives is often necessitated during such procedures. Furthermore, oxygen desaturation is known to occur at a higher frequency during flexible bronchoscopy (FB) than during other endoscopic procedures, e.g., esophagogastroduodenoscopy and colonoscopy, because of the excessive respiratory secretions and endobronchial bleeding encountered during this procedure.

Until date, the frequency of occurrence of apnea episodes during FB under sedation has never been precisely evaluated using a standardized method. Although a previous report referred to the necessity of respiratory monitoring and possible usefulness of end-tidal capnography during FB [3], supportive clinical data have not yet been obtained. The aim of this study was to investigate the actual frequency of occurrence of apnea episodes during FB under sedation by end-tidal capnography.

## Methods

This study was a retrospective review of a prospectively maintained database and medical records, including capnographic data, at our institution. This study was conducted at the Chiba University hospital (Chiba, Japan). The study protocol was submitted for Institutional Review Board approval (Chiba #2455). Written informed consent for the performance of bronchoscopy was obtained from all participants.

### Subjects

From April 2015 to March 2016, we enrolled patients who underwent diagnostic FB under midazolam sedation. The performed FB procedures included endobronchial biopsy, bronchial brushing, transbronchial biopsy, transbronchial needle aspiration (including endobronchial ultrasound-guided needle aspiration) and bronchial washing. For each patient, the following medical information was recorded from the medical records: age, gender, body mass index, American Society of Anesthesiologists (ASA) physical status classification [4], frequency of comorbidities, and final diagnosis of the lung disease.

### Sedation and patient monitoring

Midazolam was administered by intravenous bolus injection at the starting dose of 1–3 mg, with additional doses of 0.5–1 mg administered as needed for moderate sedation by pulmonologists, not anesthesiologists. Adjustment of the initial and additional doses of midazolam was left to the bronchoscopist’s discretion, guided by the criteria for moderate sedation in the ASA clinical practice guidelines [1].

Tracheal intubation was not performed. After spraying 4% lidocaine onto the patient’s throat to induce local anesthesia, a FB was inserted through the mouth of the patient lying supine on the bed. An Olympus (Tokyo, Japan) BF-260 videobronchoscope (4.9 mm outer diameter) was used. Additional local anesthesia with 1% lidocaine was used as appropriate. All patients underwent electrocardiographic monitoring and pulse oximetry as standard monitoring procedures. Supplemental oxygen (at a flow rate of 2 L/min through a nasal cannula) was used for each patient, and the oxygen flow rate was increased when the SpO_2_ fell below 90%. Decline of the SpO_2_ by ≥4% from the baseline was defined as clinically significant.

### Capnographic monitoring

**Fig. 1:**
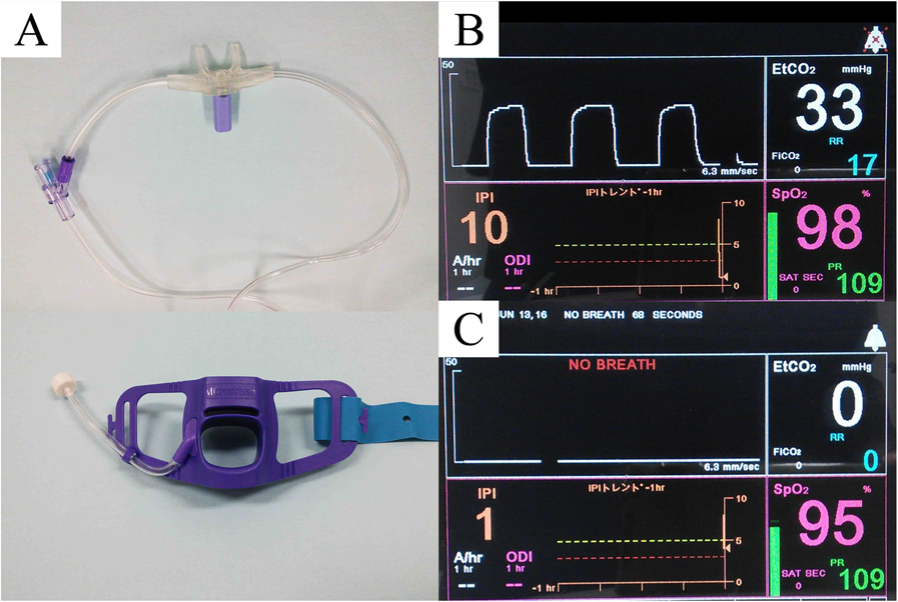
Devices used in this study for end-tidal capnography. **a** The Smart CapnoLine Guardian™ is an endoscopic bite block equipped with a cannula designed to sample expired air from both the nostril and the mouth for continuous measurement of the carbon dioxide concentration. **b** The automated capnographic monitoring device (Capnostream™ 20P) shows the patient’s respiratory airflow as a carbon dioxide concentration curve. **c** Cessation of air flow visualized as a flat line on the capnogram

For end-tidal capnographic monitoring, we used a device (Smart CapnoLine Guardian, Medtronic, Ireland) consisting of an endoscopic bite block equipped with a cannula designed to sample expired air from both the nostrils and the mouth (Fig. 1a). This was connected to an automated capnographic monitoring device (Capnostream 20P, Medtronic, Ireland) (Fig. 1b) through a sampling tube. This monitoring device can measure the concentrations of carbon dioxide in the expired air continuously. When the capnogram showed a flat line, a clinician made sure that the sample port was not disconnected. The capnographic data and SpO_2_ data were routinely collected and recorded in our institutional databank after the procedure. Apnea episodes were defined in this study as cessation of airflow for more than 10 s (Fig. 1c). After identifying both the time of onset and end of the apnea episodes, we calculated the duration of the apnea episodes and the time delay between the onset of an apnea episode and a ≥4% SpO_2_ decline from the baseline.

### Data analysis

Descriptive data were expressed as numbers (percentages) and medians (ranges), and continuous data were expressed as means and standard deviations (SD) for normally distributed data, and as medians with interquartile ranges (IQR) for non-normally distributed data. Continuous data were tested for normal distribution using the Kolmogorov–Smirnov test. For statistical comparison, the descriptive data were compared using the chi-square test or Fisher’s exact test, and the continuous data were compared using the *t*-test for normally distributed data and the Mann-Whitney test for non-normally distributed data. All *P* values were two sided and the statistical significance level was set at <0.05. Data were entered into an Excel (Microsoft Corp, USA) database. All analysis and interpretations of the data were carried out using SAS, ver. 9.4 (SAS Institute Inc. USA).

## Results

Of the 125 eligible patients, four patients were excluded because of missing data. After analysis of the capnographic data from the remaining 121 patients, the patients were divided into a group with apnea episodes (developing at least one apnea episode) and a group without apnea episodes during the FB procedure. There were 59 patients (40 males and 19 females) with a mean age of 71 years (range 23–82) in the group with apnea episodes and 62 patients (42 males and 20 females) with a mean age of 69 years (range 37–81) in the group without apnea episodes. As shown in Table 1, there were no significant differences in the age, gender, body mass index, ASA physical status, frequency of the comorbidities of COPD or interstitial lung disease, and the final diagnosis between the groups with and without apnea episodes. There were no patients with severe obesity or severe obstructive sleep apnea syndrome with abnormality of a neck circumference. The SpO_2_ was ≥96% prior to the procedure in all the patients. The mean total dose of midazolam was higher in the group with apnea episodes than in the group without apnea episodes (3.9 mg vs. 2.3 mg, *P* < 0.001). The median minimum SpO_2_ during the FB procedure was lower in the group with apnea episodes (89%; IQR 87–93 vs. 96%; IQR 92–97, *P* < 0.001) and the median maximum flow rate of supplemental O_2_ required was higher in the group with apnea episodes than in the group without apnea episodes (2 L/min; IQR 2–4 vs. 2 L/min; IQR 2-2, *P* < 0.001).

**Table 1:** Characteristics of the patient groups with and without apnea episodes (*n* = 121)

Variable	With apnea episodes (*n* = 59)	Without apnea episodes (*n* = 62)	*P* value
Age (years), median (range)	71 (23–82)	69 (37–81)	0.781
Gender (male), *n* (%)	40 (67.8)	42 (67.7)	0.995
BMI (kg/m^2^), mean ± SD	22.3 ± 2.8	21.3 ± 2.9	0.061
ASA physical status (I/II/III/IV), *n*	15/36/8/0	25/31/6/0	0.214
Comorbidities and diagnoses, *n* (%)			
COPD	1 (1.7)	4 (6.5)	0.365
ILD	11 (18.6)	8 (12.9)	0.458
Infectious disease	3 (5.1)	3 (4.8)	1.000
Malignancy	41 (69.5)	39 (62.9)	0.565
Others	12 (20.3)	11 (17.7)	0.818
Procedures, (%)			
Endobronchial biopsy	16.9	12.9	0.532
Brushing	42.4	56.5	0.122
TBB	54.2	64.5	0.250
TBNA	25.4	19.4	0.423
Washing	15.3	29.0	0.069
Baseline SpO_2_^a^ (%), mean ± SD	97.1 ± 1.0	98.0 ± 1.0	0.014
Minimum SpO_2_^b^ (%), median (IQR)	89 (87–93)	96 (92–97)	<0.001
Maximum O_2_ flow (L/min), median (IQR)	2 (2–4)	2 (2-2)	<0.001
Total dose of midazolam (mg), mean ± SD	3.9 ± 1.0	2.3 ± 0.8	<0.001

*BMI* body mass index, *SD* standard deviation, *ASA* American Society of Anesthesiologists, *COPD* chronic obstructive pulmonary disease, *ILD* interstitial lung disease, *TBB* transbronchial biopsy, *TBNA* transbronchial needle aspiration (including endobronchial ultrasound-guided aspiration), *IQR* interquartile range

^a^Before flexible bronchoscopy in ambient air

^b^During flexible bronchoscopy under supplemental oxygen

As shown in Table 2, there were a total of 131 apnea episodes in 59 patients (48.8%). Prolong apnea episodes that lasted for more than 30 s occurred in 24 patients (19.8%). The median duration of the apnea episodes was 33 s (IQR 24–46), and the maximum duration was 97 s. A total of 55 apnea episodes (42.0%) were followed by a decline of the SpO_2_ by ≥4% from the baseline. The median time delay between the onset of an apnea episode and decline of the SpO_2_ by ≥4% from the baseline was 31 s (IQR 28–42). There were no patients who suffered from massive bleeding or pneumothorax during the FB procedures. None of the patients in this case series required assisted ventilation during/after the FB procedure.

**Table 2:** Apnea profile (*n* = 131)

Apnea episodes per patient, median (range)	2 (1–8)
Duration of the apnea episodes, median (IQR)	33 s (24–46)
Maximum duration time of an apnea episode	97 s
Apnea episodes associated with SpO_2_ decline by ≥4%, n (%)	55 (42.0)
Maximum SpO_2_ decline	19% in SpO_2_
Time delay between the onset of an apnea episode and SpO_2_ decline by ≥4%, median (IQR)	31 s (28–42)

*IQR* interquartile range, *s* seconds

## Discussion

Our results revealed that apnea episodes occur at a high frequency during FB under sedation in the clinical setting. In addition, end-tidal capnographic monitoring also allowed earlier detection of the apnea episodes than pulse oximetry.

The present study showed that apnea episodes occurred frequently during FB procedures performed under sedation; apneas occurred in 48.8% of the patients, and especially prolonged apnea episodes, lasting for over 30 s, occurred in 19.8% of the patients. Moreover, as much as 42.0% of the apnea episodes were followed by significant oxygen desaturation. Previous reports may lend support to our results; in the prospective study of 50 patients undergoing colonoscopy under sedation, a total of 29 episodes of anomalous ventilation, including apnea episodes lasting for more than 30 s, were detected in 16 patients by capnographic monitoring [5]. The results of a nationwide survey reported recently from Switzerland showed that apnea episodes requiring bag ventilation occurred at a rate of 0.011% during FB under sedation [6]. However, the results of the end-tidal capnographic monitoring in our present study revealed the occurrence of a number of additional apnea episodes besides those necessitating assisted ventilation. This implies that unintended oversedation occurs in many patients sedated with midazolam in the clinical setting, even though clinicians try to titrate the level of sedation to “moderate” sedation. Moreover, the dose of midazolam used in this study might have been lower than the average dose used at other institutions. A higher number and longer length of apnea episodes can be identified by capnography in patients administered higher doses of midazolam or propofol. Therefore, we suggest that it is necessary to carry out respiratory monitoring during FB under sedation.

This study also showed that end-tidal capnographic monitoring allowed earlier detection of the apnea episodes, not actual measurement values of carbon dioxide, than pulse oximetry. It has been reported that SpO_2_ decline to its nadir can be delayed by 45 to 60 s after an apnea episode in patients with obstructive sleep apnea syndrome [7]. Our study showed that the median time delay between the onset of an apnea episode and significant SpO_2_ decline was 31 s. This suggests that we can recognize apnea episodes about 30 s earlier by capnographic monitoring than by pulse oximetry alone. This result has a very important implication in relation to the usefulness of capnographic monitoring; end-tidal capnography alerts us of apnea episodes by setting off an alarm and by way of graphics, as shown in Fig. 1c, which allows us to detect apnea episodes early and to promptly implement appropriate corrective measures, such as patient stimulation or oxygen supplementation, before the respiratory depression becomes clinically significant. Furthermore, inappropriate addition of sedatives can also be avoided by early recognition of apnea episodes. Although pulse oximetry is important to evaluate the oxygenation state of a patient and is routinely used for monitoring of respiratory functions during FB [2], it has its limitations. Pulse oximetry can be a delayed indicator of respiratory depression [8]. Especially in sedated patients receiving supplemental oxygen, the decline in the SpO_2_ may be delayed even in the presence of severe respiratory insufficiency [9, 10]. Therefore, we need an additional method for the early detection of respiratory depression. End-tidal capnography is a non-invasive method that allows continuous monitoring of ventilation. Although several studies of end-tidal capnographic monitoring during gastrointestinal endoscopy have been conducted, the conclusions remain controversial because of wide differences in the study settings (e.g. in relation to the patients’ backgrounds, the type of sedative used, the primary outcome measure, etc.) [11–13]. To investigate the usefulness of end-tidal capnography during FB, prospective comparative trials are warranted in the future.

Various methods for early detection of respiratory depression have been described so far. Monitoring of the chest wall impedance is the most commonly used monitoring method in clinical practice, and recently, the use of acoustic monitoring has also become popular in some countries. However, many factors, including motion artifacts and physiological events unrelated to respiration, such as coughing and swallowing, may generate a high number of false alarms due to inaccurate readings when these two devices are used [14–16]. Moreover, chest wall impedance monitoring may fail to detect apneas secondary to airway obstruction [17]. A previous study demonstrated that transcutaneous capnography is an effective tool for monitoring ventilation during FB [18, 19]. Cutaneous carbon dioxide tension measured with a digital sensor has been reported to show a good correlation with the arterial carbon dioxide concentration. Therefore, currently, end-tidal or transcutaneous capnographic monitoring may be the most reliable for sensitive detection of respiratory depression. However, as there have been no comparative studies to determine the best method for respiratory monitoring, further investigations are awaited.

We need to discuss some limitations of our study here. First, it is possible that flat lines on the capnogram were caused by events other than true apnea episodes. Although we checked for displacement of the sampling tube, we did not record events of continuous suctionings of the airway that may cause a cessation of the waveform on the capnogram. Second, we have to consider multiple causes of SpO_2_ decline during FB under sedation. SpO_2_ decline can also be caused by conditions such as mucus obstruction of the airways, bronchial bleeding, or pneumothorax. However, there were no cases of massive bleeding or pneumothorax in this case series, and there were no patients who underwent bronchoalveolar lavage. Therefore, most of the SpO_2_ declines are presumed to have been associated with apneic episodes in our cases.

## Conclusions

End-tidal capnography revealed the occurrence of apnea episodes at a high frequency during FB under sedation. End-tidal capnographic monitoring also allowed earlier detection of the apnea episodes than pulse oximetry. We suggest the possible necessity of respiratory monitoring during FB in the clinical setting.

## Abbreviations

ASA: American Society of Anethesiologists
FB: Flexible bronchoscopy
IQR: Interquartile ranges
SD: Standard deviations
